# Statewide Implementation of the Music & Memory Program: Facilitators, Barriers, and Lessons Learned

**DOI:** 10.1093/geroni/igaa057.194

**Published:** 2020-12-16

**Authors:** Jung Kwak, Jung-Hwa Ha, Katherine Britt

**Affiliations:** 1 University of Texas at Austin, Austin, Texas, United States; 2 Seoul National University, Seoul, Republic of Korea

## Abstract

The movement of evidence-based interventions into routine institutional settings like nursing homes is challenging. Among non-pharmacological interventions to address behavioral problems of residents with dementia, Music and Memory (M&M), a popular individualized music listening program, has been shown to have potential to improve quality of life among residents. To examine facilitators and barriers to implementation and sustainability of the M&M program in nursing facilities, a statewide (online and mail) survey of nursing homes was conducted in Wisconsin where the statewide implementation of the program occurred. The response rate was 41% (N=161). Descriptive statistics and content analysis were conducted. Over 80% of facilities provided the M&M program, and 86% of them planned to continue the program. The majority of respondents found the M&M to be beneficial to residents but also reported that the program was not equally effective for everyone, and M&M was time and labor intensive. Barriers to sustainability were: lack of buy-in by direct care staff, use of technology, costs of equipment, inconsistent volunteers, and families not supportive or helpful. Facilitators were: support of facility personnel, family, and volunteers; observing positive effects of program, M&M training provision and support, family involvement, and accessibility of equipment. Targeted resident selection is needed to identify the residents most likely to benefit from the program to avoid possibility of increased agitation or discomfort. Careful consideration is needed for facilities to identify realistic costs, labor, and staff buy-in to promote success.

